# Supplementary material for: Opinion Polls and Antibody Response Dynamics of Vaccination with COVID-19 Booster Vaccines
Source: Vaccines (Basel). 2022 Apr 20;10(5):647. doi: 10.3390/vaccines10050647 (PMC9143648; doi:10.3390/vaccines10050647)
Supplement: Supplementary file 1 [file vaccines-10-00647-s001.zip › Supplementary.pdf]

Supplementary File

Yufei Wu[1, 2, 3], Huanjie Li[1, 2,*], Yangyang Wang[1, 2], Ping Huang[2], Yihui Xu[2], Mingjie Xu[2], Qianqian Zhao[2], Yunying Zhou[2], Jun Wang[2], Mingyu Ji[1, 2, 3], and Yunshan Wang[1, 2,*]

Table S1. Linear regression model of respondent preferences with vaccination (n = 366; reference = 92.66%)

Attribute	P-value	95% CI
Age	0.691	-0.041 ~ 0.027
Education	0.984	-0.029 ~ 0.030
Occupation	0.687	-0.006 ~ 0.004
Health	0.559	-0.051 ~ 0.095
Basic illness	0.812	-0.018 ~ 0.014
Vaccine related side-effects	0.013*	-0.110 ~ -0.013
Knowledge of precautions for vaccines	0.000**	0.114 ~ 0.304
Understanding the vaccine pathway	0.597	-0.005 ~ 0.008
Knowledge of COVID-19	0.022*	0.007 ~ 0.086
Frequency of concern about COVID-19 vaccine	0.007**	0.014 ~ 0.084

* $p<0.05$, This factor has a significant correlation with the willingness to get the third dose of COVID-19 vaccine; ** $p<0.01$, This factor has a very significant correlation with the willingness to vaccinate; n = 366, 366 people who were willing to be vaccinated; reference = 92.66%, The people who are willing to be vaccinated account for 92.66% of the total number of participants.

Table S2. Symptoms by participant after the third dose of COVID-19 vaccine (n=138)

Individual symptoms	n	%
No adverse effect	78	56.52%
Dizziness and nausea	7	5.07%
Fever	7	5.07%
Muscle ache	44	31.88%
Cough	1	0.72%
Diarrhea	1	0.72%
got the third dose of COVID-19 vaccine	138	

n = 138, 138 people who have the third dose of COVID-19 vaccine; %, the percentage of this item to the total number of 138.

Table S3. Analysis of respondents' awareness of the necessity of the third shot (booster shots)

Attitude	Necessity of Getting booster shots of COVID-19 Vaccine(n=395)									
	Extremely disapproval		Disapproval		Unsure		approval		Extremely approval	
	n	%	n	%	n	%	n	%	n	%
Significance	11	2.78	7	1.77	44	11.14	58	14.68	275	69.62
Preventability	13	3.29	9	2.28	43	10.89	55	13.92	275	69.2
Protective Capability	14	3.54	7	1.77	43	10.89	54	13.67	277	70.13
Necessity	13	3.29	10	2.53	38	9.62	44	11.14	290	73.42

n. The total number of people who participated in the survey; %, the percentage of this item to the total number of participants.

Table S4. SARS-CoV-2 S-specific binding antibody spearman correlation (detail)

		SARS-CoV-2 S-specific binding Antibody 7 days after vaccination	SARS-CoV-2 S-specific binding Antibody 14 days after vaccination	SARS-CoV-2 S-specific binding Antibody 21 days after vaccination	SARS-CoV-2 S-specific binding Antibody 28 days after vaccination	SARS-CoV-2 S-specific binding Antibody 56 days after vaccination
SARS-CoV-2 S-specific binding Antibody 7 days before vaccination	Coefficient	0.4	0.8**	0.7**	0.7**	0.5*
	p value	0.2	0.0	0.0	0.0	0.0
Time interval between last needle and strengthening needle	Coefficient	0.1	-0.3	-0.3	-0.2	-0.3
	p value	0.7	0.2	0.2	0.4	0.2

* p<0.05 ** p<0.01

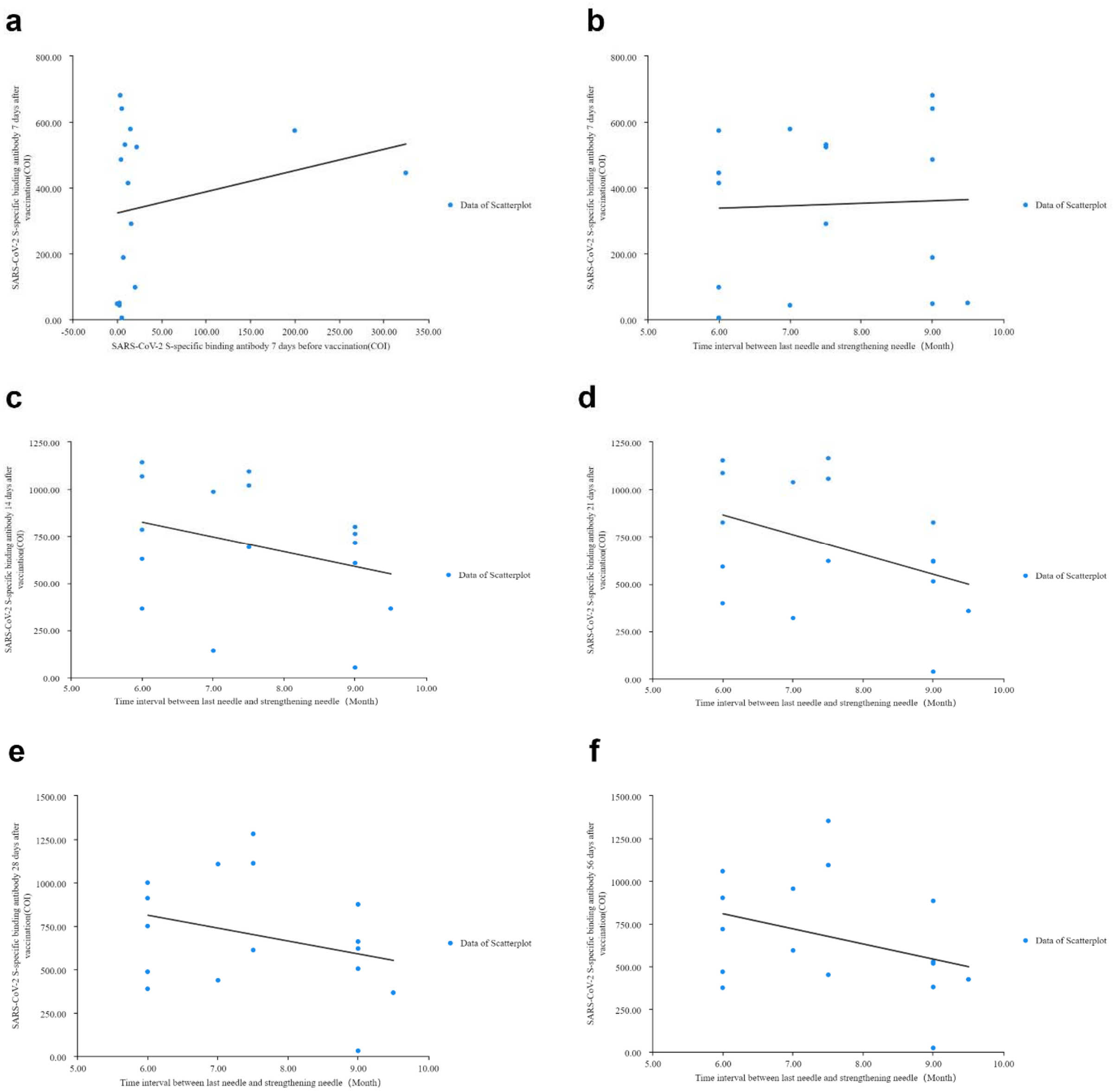

Figure. S1. The correlation analysis initial antibody concentration or booster vaccination interval and antibody concentration.

a. The correlation between initial antibody concentration and antibody concentration at seven days after vaccination.

b-f. The correlation between booster vaccination interval and antibody concentration at seven days after vaccination.

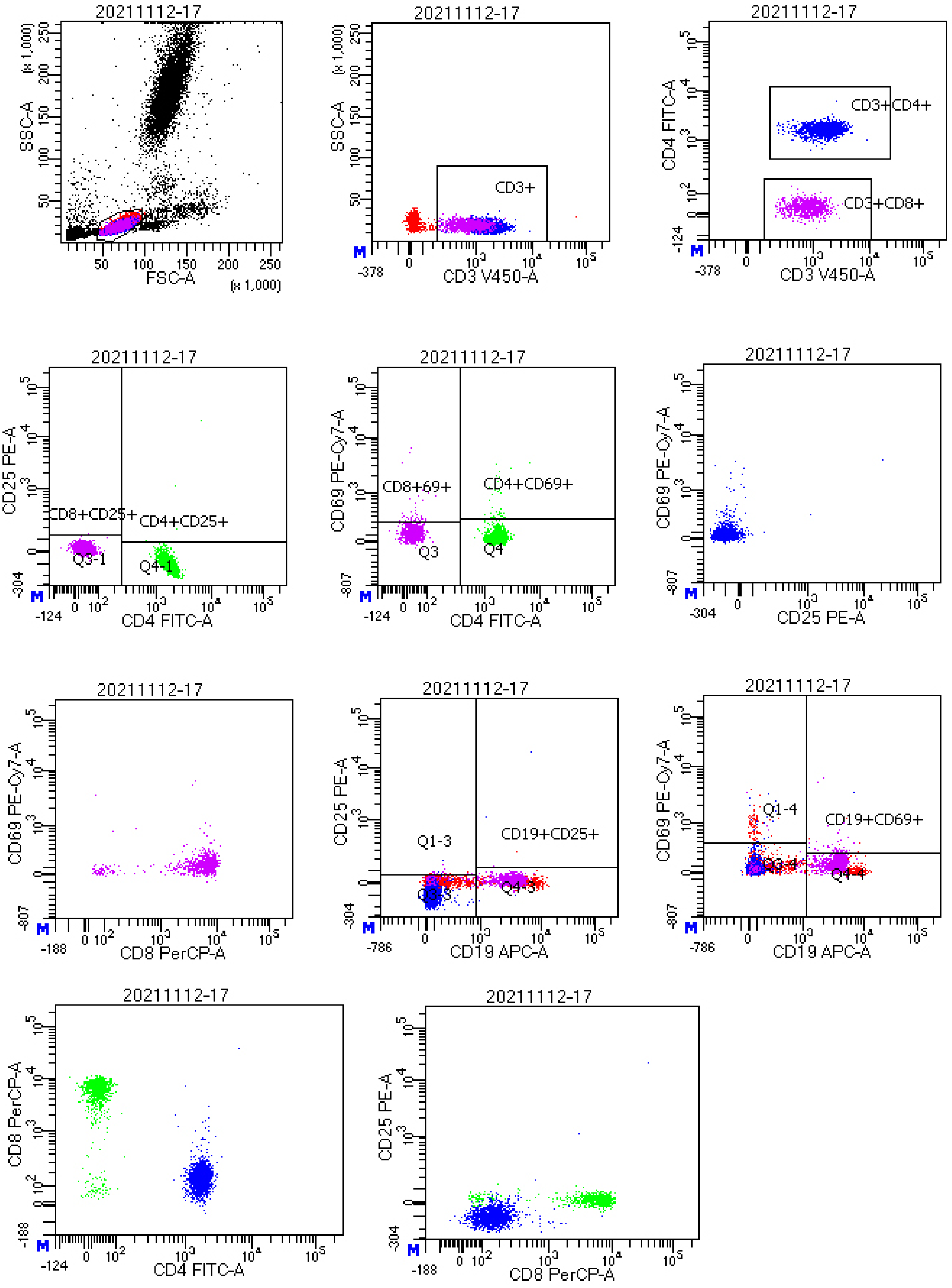

Figure. S2. CD4+, CD8+, CD25+, CD69+ cells responses to COVID-19 vaccination. Intracellular cytokine staining T-cell responses including CD4, CD8, CD69 and CD25.

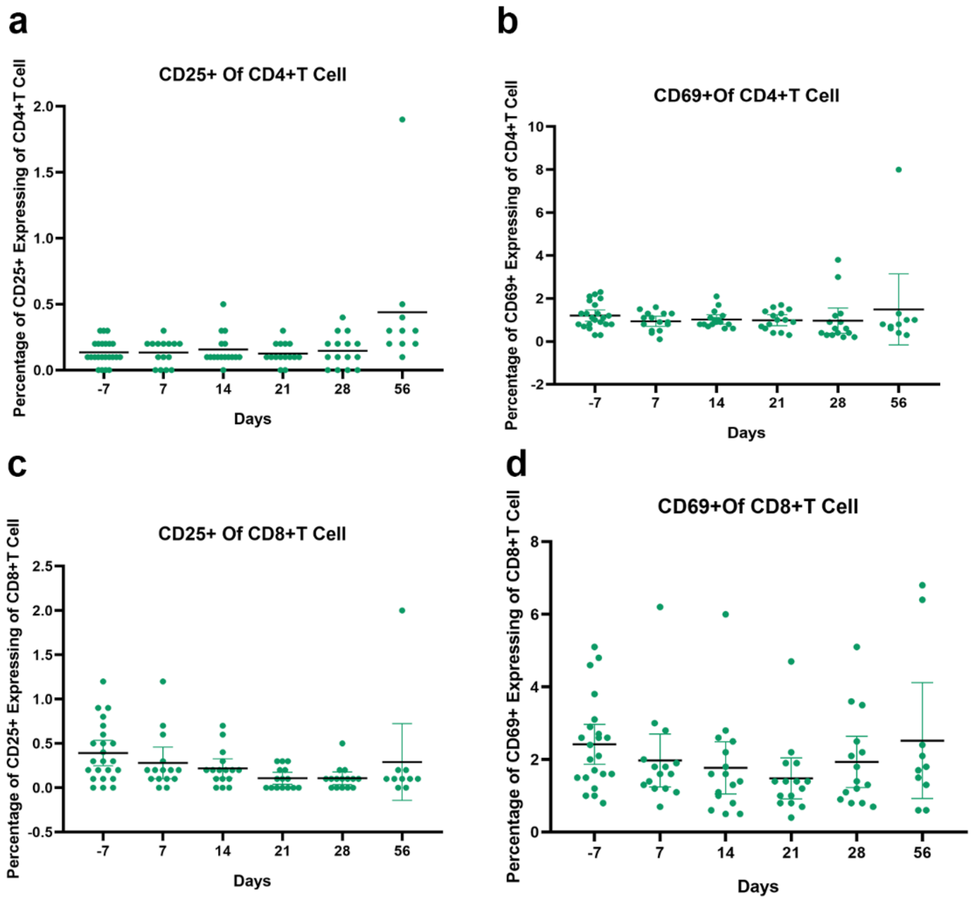

Figure. S3. CD25+, CD69+ of T cell responses to COVID-19 vaccination of 16 volunteers.

a. CD25+CD4+ T cell trend chart of the time points before and after COVID-19 vaccination;

b. CD69+CD4+ T cell trend chart of the time points before and after COVID-19 vaccination;

c. CD25+CD8+ T cell trend chart of the time points before and after COVID-19 vaccination;

d. CD69+CD8+ T cell trend chart of the time points before and after COVID-19 vaccination;
